# Development of a Liposome Nanoformulation for the Delivery of Lipoic Acid as a Potential Neuroprotective Therapy in Glaucoma

**DOI:** 10.3390/pharmaceutics17050664

**Published:** 2025-05-18

**Authors:** Pablo Edmundo Antezana, Ailen Gala Hvozda Arana, Sofia Municoy, Martín Federico Desimone, Pablo Evelson, Sandra Ferreira

**Affiliations:** 1 CONICET-Universidad de Buenos Aires, Instituto de Bioquímica y Medicina Molecular (IBIMOL), Buenos Aires 1113, Argentina; pabloantezana@ffyb.uba.ar (P.E.A.); agala@docente.ffyb.uba.ar (A.G.H.A.); 2 Universidad de Buenos Aires, Facultad de Farmacia y Bioquímica, Departamento de Ciencias Químicas, Cátedra de Química Analítica Instrumental, Buenos Aires 1113, Argentina; smunicoy@docente.ffyb.uba.ar (S.M.); desimone@ffyb.uba.ar (M.F.D.); 3 Universidad de Buenos Aires, Facultad de Farmacia y Bioquímica, Departamento de Ciencias Químicas, Cátedra de Química General e Inorgánica, Buenos Aires 1113, Argentina; 4 CONICET-Universidad de Buenos Aires, Instituto de Química y Metabolismo del Fármaco (IQUIMEFA), Buenos Aires 1113, Argentina; 5 Instituto de Ciências Biológicas (ICB), Universidade Federal do Rio Grande—FURG, Rio Grande 473, Brazil

**Keywords:** liposome, lipoic acid, glaucoma, nanoformulation, neuroprotection

## Abstract

**Background/Objectives**: Glaucoma is the leading cause of irreversible blindness worldwide and oxidative stress is considered to play a key role in its development. While antioxidants offer a promising approach to mitigating oxidative stress, their clinical application is often hindered by bioavailability and absorption challenges. Entrapment antioxidants within liposomes may overcome these issues, enhancing their stability and delivery. The aim of this study was to develop a novel composite liposomal formulation for glaucoma treatment, designed to enhance lipoic acid bioavailability and administration through its incorporation into the lipid bilayer. **Methods**: Liposomes were prepared via lipid film hydration and extrusion. To characterize them, the following analyses were performed: FTIR spectroscopy, liposomal bilayer melting temperature (Tm), TEM, DLS, Z-potential, antioxidant activity, and cytotoxicity assays. **Results**: The efficient incorporation of lipoic acid into the liposomes’ lipid bilayer was confirmed by FTIR. This incorporation resulted in an increase in the Tm, from 37.0 °C for liposomes to 40.0 °C for liposomes with lipoic acid (L-LA). TEM images confirmed that the spherical morphology of the lipid vesicles remained unchanged following LA incorporation. Dynamic Light Scattering analysis revealed effective diameters of 423 ± 36 nm for L liposomes and 404 ± 62 nm for L-LA liposomes. Notably, the Z-potential shifted from +4.7 ± 0.4 mV (L) to −0.4 ± 0.3 mV (L-LA). Furthermore, L-LA exhibited significant antioxidant activity (31.6 ± 0.4%) compared with L (5.3 ± 0.3%) and biocompatibility, suggesting its potential for therapeutic applications. **Conclusions**: In summary, biocompatible composite liposomes with antioxidant capacity were successfully developed, resulting in promising candidates for neuroprotective glaucoma therapy.

## 1. Introduction

Glaucoma is the leading cause of irreversible blindness worldwide, characterized by a pattern of damage to the optic nerve, which causes visual field abnormalities and the death of retinal ganglion cells (RGCs) [1,2,3]. Nowadays, this pathology is considered a neurodegenerative disease since the atrophy of retinal ganglion cells and axonal degeneration spread to the brain’s central visual targets through transsynaptic degeneration [4,5,6,7]. Among the proposed physiopathological damage mechanisms, oxidative stress is considered to play a key role in the development of glaucoma. Oxidative stress is characterized by an imbalance between oxidants and antioxidants, in favor of the former, potentially disrupting metabolic signaling and transcription processes and causing oxidative damage to macromolecules [8]. It has been proposed that it may not only affect ocular structures but also brain areas related to vision [9,10,11,12,13]. Furthermore, evidence indicates that oxidative stress also plays a significant role in corneal damage in glaucoma [14].

The primary objective of glaucoma treatment is the reduction in intraocular pressure (IOP) to mitigate progressive retinal ganglion cell loss and irreversible optic nerve damage. Initial therapeutic strategies typically involve eye drops with topical pharmacologic agents, such as prostaglandin analogs, beta-blockers, alpha-adrenergic agonists, and carbonic anhydrase inhibitors. If these strategies are not sufficient to control the IOP, laser and/or surgical interventions are used [15]. Alternatively, treatments designed to prevent neuronal death in glaucoma are known to act as neuroprotectors [16]. Antioxidants are an interesting tool for treating/preventing the consequences of the occurrence of oxidative stress, as has been shown in other diseases such as cataracts [17]. However, their administration can be complex due to factors such as bioavailability and absorption. For this reason, designing liposomes that contain antioxidants could improve their efficacy [18].

Liposomes can be described as a spherical structure with a membrane made up of phospholipid bilayers, similar to the composition of cellular membranes. These structures present a hydrophobic membrane and an aqueous core. This dual composition enables liposomes to encapsulate both hydrophilic (in their aqueous core) and lipophilic (in their hydrophobic lipid bilayer) drug molecules, making them highly versatile as drug delivery systems [19]. Among their advantages, it is worth mentioning their effectiveness as a promising vehicle for drug delivery and targeting, due to their non-toxicity, biodegradability, biocompatibility, and their ability to modify their surface and size [20,21]. A major drawback of liposomes is their high production cost and a possible short half-life in the bloodstream. However, liposomes are still an interesting alternative tool for improving the release of active compounds since modifications could be made to their structure to improve their half-life [22]. Regarding the use of liposomes in the treatment of ophthalmic diseases, studies have shown that encapsulated drugs are able to cross the corneal epithelium due to the solubilization of liposomes in cellular lipid membranes [23].

Among the antioxidants studied in glaucoma pathology, lipoic acid has shown an interesting role, as its oral administration improves the activation of antioxidant genes and proteins expression, leading to the protection of RGCs and the prevention of oxidative damage (evaluated as a decrease in lipid peroxidation, protein nitrosylation, and DNA oxidation in the retina) [24]. Moreover, it has been proved that lipoic acid exhibits a potent neuroprotective effect against oxidative stress-induced cell death in the retinal neuronal RGC-5 cell line promoting the nuclear translocation of the Nrf2 factor, thereby enhancing the expression of heme oxygenase-1 (HO-1) [25]. In this vein, lipoic acid is a key antioxidant since it does not affect the intraocular pressure due to its several action mechanisms—such as chelating redox-active transition metals to inhibit the formation of hydrogen peroxide and hydroxyl radicals, scavenging reactive oxygen species, downregulating inflammatory processes, increasing Nrf2 nucleus translocation and the neutralization of lipid peroxidation products inducing the synthesis of glutathione and other antioxidant protective enzymes—that reinforce its potential neuroprotective effects [26]. However, among the disadvantages that lipoic acid might present, it should be mentioned that it is not water-soluble and is vulnerable to oxidation, making it an unstable molecule [27]. It has been shown that lipoic acid could be incorporated into liposomes formulations in order to decrease the toxicity of chemotherapeutic drugs [28]. In addition, it has been proved that the inclusion of lipoic acid in liposomes enhanced its stability, absorption, and bioavailability with hepatoprotective effects [29]. Phosphatidylcholine-curcumin liposomes encapsulating lipoic acid have been used to reduce cisplatin-induced ototoxicity, with a high encapsulation efficiency and sustained release [30]. Considering these previous outcomes in the development and application of liposomes in different diseases, it is reasonable to hypothesize that encapsulating lipoic acid in liposomes could enhance its stability and release, leading to the development of a topical treatment that provides neuroprotection for RGCs. Therefore, the aim of this study was to develop a novel formulation for topical glaucoma treatment that enhances the bioavailability upon the administration of lipoic acid by incorporating it into the lipid bilayer of liposomes. In order to achieve this purpose, composite liposomes composed of 1,2-dipalmitoyl-sn-glycero-3-phosphatidylcholine (DPPC), 1-palmitoyl-2-oleoyl-sn-glycero-3-phosphatidylcholine (POPC), and lipoic acid were synthesized and characterized.

## 2. Materials and Methods

### 2.1. Synthesis of Liposomes and Incorporation of Lipoic Acid

**Scheme 1:**
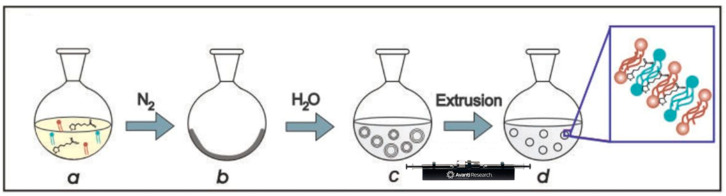
Scheme describing the synthesis of liposomes with lipoic acid. (**a**) DPPC, POPC, and lipoic acid were dissolved in chloroform; (**b**) a lipid film was formed under a N_2_ flow; (**c**) the dry lipid film was hydrated above Tm of the lipids (50 °C) by adding 1 mL of sterile saline solution, obtaining a suspension of multilamellar liposomes of different sizes; (**d**) liposomes were homogenized by multiple extrusions at 50 °C through polycarbonate membrane filters of decreasing pore diameters of 800 and 400 nm.

Liposomes (L) were prepared by the lipid film hydration and extrusion method [21,22]. For this, 7 mg of 1,2-dipalmitoyl-sn-glycero-3-phosphatidylcholine (DPPC) (850355P, Avanti Research), 2 mg of 1-palmitoyl-2-oleoyl-sn-glycero-3-phosphatidylcholine (POPC) (850457P, Avanti Research, Alabaster, AL, USA) [21,22], and 5 mg of lipoic acid (LA) (T5625, Sigma Aldrich, St Louis, MO, USA), based on previous studies performed with lipoic acid in retinal ganglion cell cultures [25], were first dissolved in chloroform and then dried under a nitrogen stream. The dry lipid film was then hydrated above the main phase transition temperature (Tm) of the lipids (50 °C) by adding 1 mL of sterile saline solution. The obtained liposomes (L-LA) were homogenized by multiple extrusions at 50 °C through polycarbonate membrane filters (Avanti Mini Extruder, Avanti Research, Alabaster, AL, USA) of decreasing pore diameters of 800 and 400 nm. Empty liposomes were prepared following the same method but without the addition of lipoic acid during film formation (Scheme 1).

### 2.2. Characterization

Fourier transform infrared (FTIR) spectra of pure LA, L-LA, and empty liposomes were obtained over the range of 4000–500 cm^−1^, using an FTIR-Raman Nicolet iS 50 (Thermo Fisher Scientific, Waltham, MA, USA). For this, an aliquot of the samples was dried under a nitrogen flow and the powder was then placed on the attenuated total reflection accessory of the spectrometer and pressed to record the spectra. The phase transition temperature (Tm) of the lipid membrane was determined by turbidimetry, measuring the variation in intensity of the light transmitted through the liposome suspension as a function of temperature (20–50 °C) using a UV–visible spectrophotometer (Varian Cary 50 UV–Vis, Agilent Technologies, Santa Clara, CA, USA). The obtained sigmoid curve was then fitted to the sigmoidal dose–response model and Tm was determined at the inflection point of the curve. The morphology of L and L-LA was studied by transmission electron microscopy (TEM) using a Zeiss EM109T electron microscope. The samples for TEM were negatively stained with uranyl acetate 2% and a drop of this was added to carbon–copper grids and allowed to dry for a few minutes. Size distribution and surface charge of L and L-LA was determined by Dynamic Light Scattering (DLS) and Z-potential measurements using a Zetasizer Nano-Zs (Malvern Instruments, Malvern, UK), equipped with a He–Ne laser (633 nm) and a digital correlator model ZEN3600.

### 2.3. Quantification and Release Study of LA

LA was quantified by high-performance liquid chromatography. For this, a Waters Alliance 2695 HPLC with a Waters 2996 Photodiode Array Detector was used. The analytical column was a C18, 250 × 4.6 mm ID, 5µ particle size (Agilent Technologies, Santa Clara, CA, USA). The mobile phase comprised 50 mM potassium dihydrogen phosphate (pH 4.5 adjusted with 1 M H_3_PO_4_) and acetonitrile in the ratio of 50:50. The mobile phase was filtered through microporous membrane filters with a pore size of 0.2 µm to remove particulate impurities and then sonicated for 10 min to remove dissolved gas. The UV detector was set at 245 nm. The sample (10 μL) was injected into the HPLC system and the chromatogram was run for 10 min at a flow rate of 1 mL/min.

Stock standard solution of 5 mg/mL of LA was prepared by dissolving the pure powder in absolute ethanol. Then, a calibration curve was prepared by including standard concentrations of 0.5, 1.0, 2.5, and 5 mg/mL of LA.

For the quantification of LA in the liposomes and to determine the entrapment efficiency, L-LA was lysed. For this, only the suspension of L-LA was collected (avoiding any precipitate at the bottom corresponding to insoluble and non-entrapped LA), diluted four times with absolute ethanol and disrupted by sonication for 2 h [31,32]. Then, 10 μL of the obtained suspension was injected into the HPLC system and the chromatogram was run for 10 min at a flow rate of 1 mL/min.

Once the entrapment efficiency in the liposomes was determined, the release study was conducted under two different conditions. To study the release of LA from the liposomes, 0.2 mL of L-LA was diluted with 1.8 mL of saline solution. One milliliter of the mixture was incubated at 40 °C, while the other was kept at room temperature. After 24 h, both suspensions were centrifuged at 10,000 rpm for 10 min, and 10 μL of the supernatants was injected into the HPLC system and the chromatogram was run for 10 min at a flow rate of 1 mL/min. The encapsulated concentration was considered 100%, and the amount released was expressed relative to this value, using the following equation:

$$\%\;\mathrm{LA}\;\mathrm{Release}=\frac{\mathrm{Release}\;\mathrm{concentració}n}{\mathrm{Encapsulated}\;\mathrm{concentracion}}\times 100$$

Results are expressed as mean ± SD from triplicate experiments.

### 2.4. Antioxidant Activity

The antioxidant activity of L, L-LA, and LA (5 mg/mL) was evaluated by the DPPH colorimetric assay [33,34], which measures the scavenging activity of the 2,2-diphenyl-1-picrylhydrazyl free radical (DPPH•). For this, 2 mL of a methanolic solution of DPPH• (25 mg/L) was added to 500 μL of L, L-LA, and LA and incubated for 5 min at room temperature. Finally, absorbance was measured at 515 nm and antioxidant capacity was calculated as a percentage of inhibition using the following equation:

$$\%\;\mathrm{Inhibition}=\left[1-\left(\frac{\mathrm{Abs}\;\mathrm{sample}}{\mathrm{Abs}\;\mathrm{DPPH}\;\mathrm{solution}}\right)\right]\times 100$$

Results are expressed as mean ± SD from triplicate experiments.

### 2.5. Cytotoxicity Evaluation

Healthy human conjunctival epithelium cell line (IOBA-NHC) cells were used to study the cytotoxicity of L and LA. The IOBA-NHC cell line was provided by Yolanda Diebold, PhD (University Institute of Applied Ophthalmobiology, University of Valladol- id, Valladolid, Spain). The cells were cultured in a humidified chamber (95% air; 5% CO_2_) at 37 °C in Dulbecco’s Modified Eagle’s Medium-F12 (DMEM-F12) and supplemented with 10% fetal calf serum and 1% penicillin–streptomycin. Once in confluence, cells were trypsinized and counted using a Neubauer camera. A total of 1 × 10^4^ cells were seeded with 1000 μL of DMEM in a 24-well plate and were incubated at 37 °C for 24 h. A colorimetric 3-(4,5-dimethyl-thiazol-2-yl)-2,5-diphenyl-tetrazolium bromide (MTT) assay was used to evaluate the LA cytotoxicity. After 24 h, we removed the medium, added 0.5 mL of MTT solution (5 mg/mL) and incubated the mixture for 3 h at 37 °C. Then, the MTT solution was discarded and 0.5 mL of absolute ethanol was added. The absorbance values were measured at 570 nm and results are expressed as mean ± SD from triplicate experiments.

### 2.6. Statistical Analysis

For statistical analysis GraphPad Prism 5.0 software (GraphPad Software, La Jolla, CA, USA) was used. Data were expressed as the mean ± SD. The statistical significance of the differences between the groups was calculated using a one-way ANOVA test followed by Bonferroni’s test as a post hoc test. A *p*-value *<* 0.05 was considered statistically significant.

## 3. Results and Discussion

### 3.1. Characterization of Liposomes

**Figure 1:**
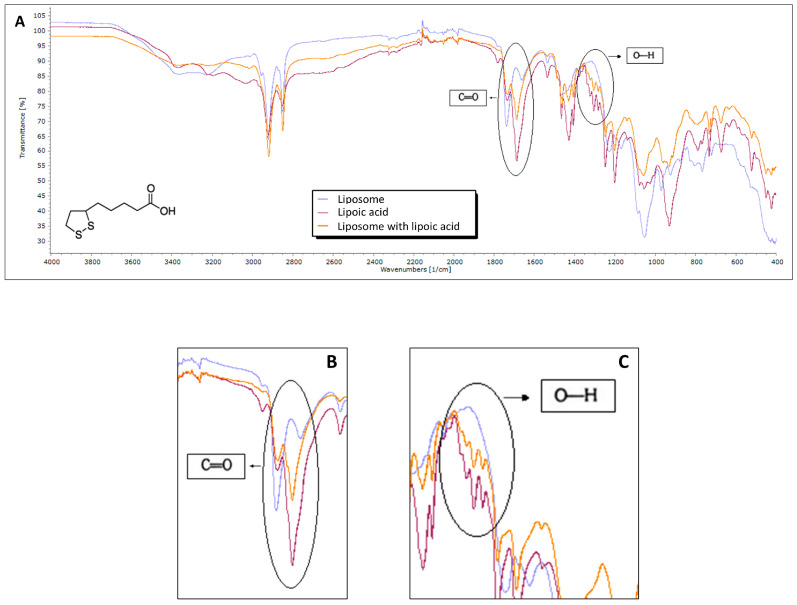
(**A**) Fourier transform infrared spectroscopy (FTIR) of liposomes (L, blue), lipoic acid (LA, red), and liposomes with lipoic acid (L-LA, yellow). (**B**) Inset of spectrum peaks at 1700 cm^−1^, corresponding to the carbon–oxygen double bond. (**C**) Inset of spectrum peaks at 1330–1400 cm^−1^, corresponding to the oxygen–hydrogen interaction.

Liposomes were prepared by the lipid film hydration and extrusion method [21,22]. After the liposome synthesis, the presence of lipoic acid was studied by comparing the FTIR spectra of empty liposomes, lipoic acid, and liposomes containing lipoic acid. In the L-LA spectrum, two characteristic peaks were observed at 1700 cm^−1^ and 1330–1400 cm^−1^, corresponding to the carbon–oxygen double bond and the oxygen–hydrogen interaction, respectively. In addition, a broad band was observed between 3400 and 3200 cm^−1^, characteristic of the hydroxyl (O–H) group, especially in lipoic acid and in the liposome containing lipoic acid. An increase in intensity and/or broadening in the liposome with lipoic acid suggests possible hydrogen bond formation upon acid incorporation. Changes in the region of 1600–1400 cm^−1^ may be related to C=C or C–O bond interactions, or CH_2_/CH_3_ deformation modes. Variations between the spectra suggest molecular interactions between lipoic acid and the liposome components. The region of 3000–2800 cm^−1^ displays aliphatic C–H stretching bands, typical of lipids and liposomal structures. These bands remain similar across all three spectra, indicating that this region is not significantly affected by the incorporation of lipoic acid. Marked differences are observed in the 1300–900 cm^−1^ region, especially between the plain liposome and the liposome containing lipoic acid. These differences further support the occurrence of chemical or physical interactions between the two components and show that liposomes were efficiently incorporated into the lipid bilayer of the liposomes (Figure 1).

**Figure 2:**
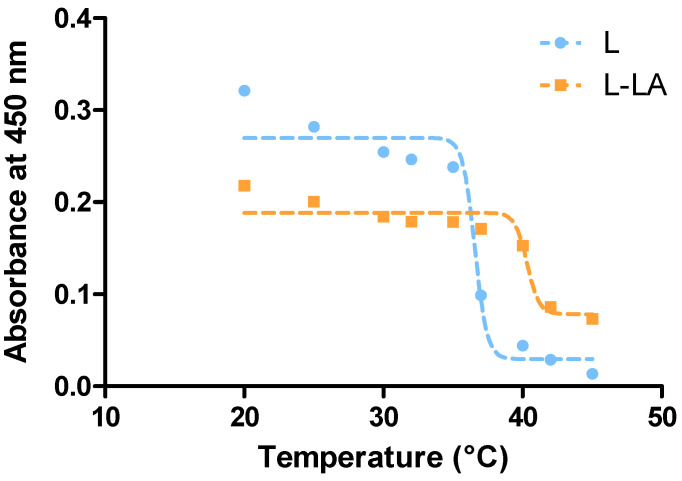
Phase transition temperatures (Tm) of liposome (L) and liposomes with lipoic acid (L-LA) determined by turbidimetric analysis.

The phase transition temperature (Tm) of the lipid bilayer is an important factor throughout the development of the liposomes, since it influences liposome permeability and regulates their release properties. In this context, it is important to consider the ocular surface temperature during the liposome’s preparation since the aim of this study is the design of potential eye drops to be used topically in glaucoma pathology. In this sense, liposomes were prepared using the lipid film hydration and extrusion method with DPPC (43 °C), POPC (−2 °C), and lipoic acid [35]. The Tm of L and L-LA liposomes formulations were then determined via turbidimetry [36]. Tm values were determined using the sigmoidal dose–response model and were found to be 37.0 °C for L and 40.0 °C for L-LA (Figure 2). These results indicate that the inclusion of lipoic acid leads to an increase in the lipid bilayer’s Tm. This is an interesting fact, since different authors have shown that the ocular surface temperature is increased by approximately 1° in glaucomatous eyes, with the mean of the medial zone being nearly 32.0° C [37]. A Tm below ocular surface temperature would mean an abrupt release of lipoic acid due to the high permeability of liposomal membranes. Thus, it was important to select phospholipids that would allow us to obtain a membrane with a Tm above glaucomatous eye temperature, to control the release of LA. In this context, the synthesized L-LA are stable at that temperature preventing the non-controlled release of drugs and achieving a more controlled action in glaucomatous patients.

**Figure 3:**
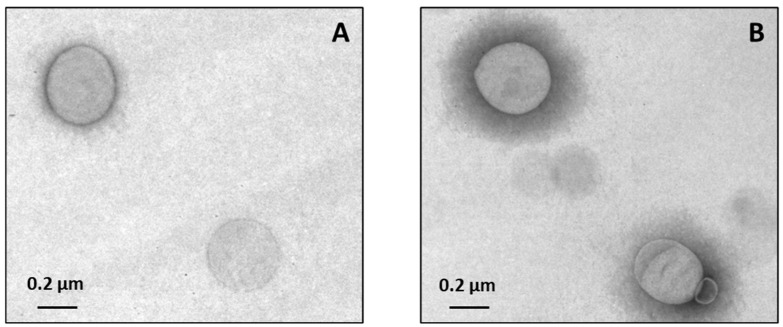
Transmission electron microscopy (TEM) image of liposomes. (**A**) Representative image of liposomes (L) shows its spherical morphology. Scale bar is 0.2 µm. (**B**) Representative image of liposomes with lipoic acid (L-LA) shows that liposomes conserve their spherical structure. Scale bar is 0.2 µm. Digital zoom from images with magnification: 12,000× (**A**) and 20,000× (**B**).

**Figure 4:**
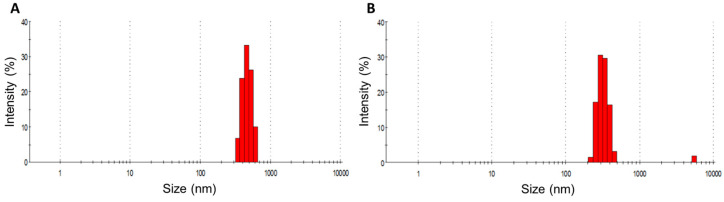
Dynamic Light Scattering (DLS) shows the size distribution of (**A**) liposomes and (**B**) liposomes with lipoic acid (L-LA).

In order to develop a new ophthalmic drug delivery system, the drug size is crucial. In this sense, particles larger than 10 μm cannot be absorbed by ocular tissues or eliminated through the nasolacrimal duct, potentially leading to ocular irritation [38]. During this characterization, transmission electron microscopy and laser light scattering were used to study morphology and size. TEM images of empty liposomes (Figure 3A) and liposomes loaded with lipoic acid (Figure 3B) confirm that the spherical shape of the lipid vesicles remained unchanged after the incorporation of LA, since the diameters obtained were 440 ± 74 and 428 ± 75 nm for liposome (L) and liposome with lipoic (L-LA), respectively. In addition to the TEM results, the DLS analysis confirmed that the effective diameter of L and L-LA was 423 ± 36 nm and 404 ± 62 nm, respectively (Figure 4A,B). The polydispersity index (PDI) of the developed liposomes was 0.157 ± 0.070 for L and 0.310 ± 0.064 for L-LA. These results indicate a good distribution since in lipid-based drug delivery systems, such as liposomes, a PDI of 0.3 or lower is generally acceptable because it reflects a uniform distribution of phospholipid vesicles [39]. These results show that the incorporation of lipoic acid did not significantly modify the structure and the size of the liposomes. In addition to this, the size of the synthetized liposomes is suitable for use as a drug delivery system in ophthalmic diseases.

**Figure 5:**
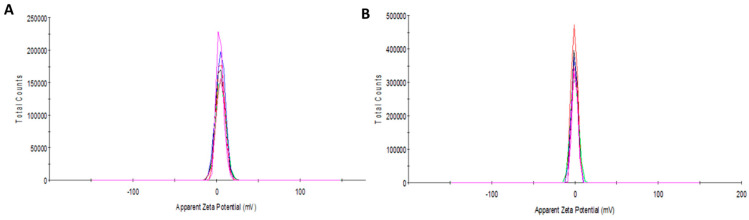
Z-potential analysis of (**A**) liposomes and (**B**) liposomes with lipoic acid (L-LA). The different colors correspond to different measurements of the same sample to demonstrate reproducibility.

Additionally, the liposomes were found to be neutral as the Z-potential of L and L-LA was +4.7 ± 0.4 mV and −0.4 ± 0.3 mV, respectively (Figure 5). Since the corneal surface presents a negatively charged mucin layer, a neutral formulation could be used as a drug delivery system for ophthalmic pathologies. It has been shown that liposomes loaded with timolol have better retention in the precorneal area compared with timolol eye drops [40]. In this sense, considering the results of both the size and surface charge of the liposomes with lipoic acid, it is reasonable to hypothesize that they could be used as a topical treatment on the ocular surface. The size allows the absorption of the liposomes by the ocular surface and since it is not negatively charged it would not present repulsion by the mucin layer of the ocular surface.

### 3.2. Quantification and Release Study of LA

The concentration of lipoic acid-loaded liposomes was determined by high-performance liquid chromatography (HPLC), resulting in a value of 4.08 ± 0.03 mg/mL. Based on this measurement, the encapsulation efficiency was calculated to be 82%.

The release profile of lipoic acid was subsequently evaluated under two different conditions: at room temperature and at the lipid phase transition temperature. The cumulative release at room temperature was 91.75%, while at the transition temperature it reached 94.3%.

These results show the successful incorporation of lipoic acid into liposomes, as indicated by an encapsulation efficiency of 82%, demonstrating the effectiveness of the preparation method employed. The high encapsulation rate suggests that the formulation parameters were well optimized to favor the retention of the active compound within the lipid bilayer. Additionally, the release studies showed that a significant part of the encapsulated lipoic acid was released under both tested conditions, with slightly higher release observed at the lipid phase transition temperature. This behavior is consistent with the expected increase in membrane fluidity during the phase transition, which facilitates the diffusion of the encapsulated drug. These results support the potential use of the liposomes with lipoic acid in therapeutic applications, as it ensures that lipoic acid remains protected within the liposomes until release, thereby preserving its biological activity.

### 3.3. Antioxidant Activity

Following characterization, the antioxidant activity of liposomes was tested using the 2,2-diphenyl-1-picrylhydrazyl (DPPH•) free radical assay. Table 1 shows that the lipoic acid solution did not display high antioxidant activity. This could be associated with the mechanism of lipoic acid, since it is described that although the antioxidant effects of lipoic acid are primarily attributed to its ability to counteract reactive oxygen species, an alternative mechanism suggests that lipoic acid may exert its effects by generating reactive sulfur species, such as hydrogen sulfide and polysulfides, which possess significant antioxidant properties, including the activation of the Nrf2 pathway [41]. However, the inclusion of lipoic acid in liposomes demonstrated enhanced antioxidant activity, likely due to its improved availability to the DPPH radical, facilitated by its adsorption onto the lipid membrane. Based on these results, it can be concluded that composite liposomes with an antioxidant capacity have been successfully synthesized, making them promising candidates as a neuroprotective therapy for glaucoma.

### 3.4. In Vitro Studies of Liposomes Biocompatibility

**Figure 6:**
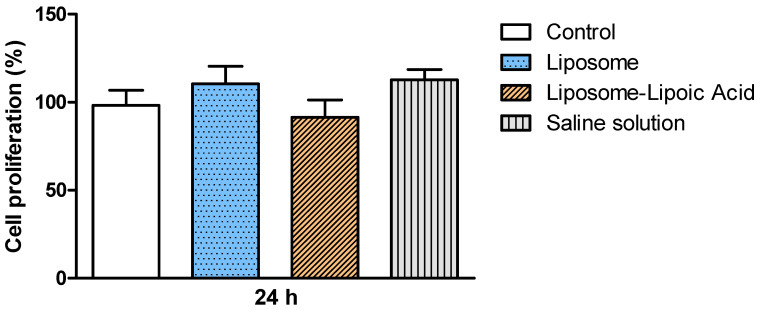
Viability of IOBA-NHC cells evaluated by the MTT test at 24 h for liposomes (L) and liposomes with lipoic acid (L-LA). Viability rate in control was considered as 100%. Results are expressed as mean ± SD from triplicate experiments. Statistical significance of the differences among groups was calculated by one-way ANOVA test followed by Bonferroni’s test as a post hoc test.

Healthy human conjunctival epithelium cell line (IOBA-NHC) cells were used to study the cytotoxicity of L and L-LA by the MTT method. Figure 6 shows that both liposomes and liposomes with lipoic acid allow the normal growth of IOBA cells. This could be attributed to the high biocompatibility of liposomes, showing that the lipoic acid did not affect the cellular development [21,42]. These results are promising since the liposome nanoformulation did not alter the growth of ocular surface cells, demonstrating that it could be used as a topical treatment in the form of ophthalmic eye drops.

## 4. Conclusions

Currently, glaucoma is considered a neurodegenerative disease and, since oxidative stress is considered to play a key role, antioxidants could be used as a novel therapeutic approach. In this study, we developed a novel formulation for glaucoma treatment that enhances the bioavailability and administration of lipoic acid by incorporating it into the lipid bilayer of liposomes. In order to assess this purpose, liposomes with a specific transition temperature were designed and synthesized using two different types of phosphatidylcholines (DPPC and POPC). Lipoic acid is a molecule with multiple benefits and growing interest in the biomedical field. However, its limited solubility represents a significant challenge that restricts its therapeutic application. Indeed, lipoic acid has low solubility in water (around 0.24 g/L) and a log *p* value of approximately 2.1. A log *p* value of 2.1 indicates that the *p* value is 10^2.1^, or approximately 126, indicating that at equilibrium, lipoic acid is 126 times more concentrated in octanol (the lipid phase) than in the water phase, demonstrating a greater affinity for lipids than water. In this context, its incorporation into liposomal membranes emerges as a promising strategy to enhance its bioavailability and expand its potential in various biomedical applications. In addition, DPPH assay allowed us to evaluate the total charge of lipid-soluble antioxidants, which is considered the first line of defense against lipid-peroxidation, protecting the cell membrane at the early stage of free radical damage. The lipid-soluble antioxidants measured by DPPH may be one of the main reasons responsible for the lipid-peroxidation inhibition. In addition, the designed composite liposomes allowed the proliferation of the conjunctival cells, showing interesting biocompatibility.

Moreover, the liposomes’ aqueous core is available to encapsulate various therapeutic molecules, leading to the possibility of the creation of versatile nanomaterials with broad clinical applications. Future investigations should focus on conducting in-depth, in vivo ocular retention studies, utilizing experimental models of ocular hypertension to evaluate pharmacological efficacy, and performing a thorough characterization of the pharmacokinetics of lipoic acid within the different ocular compartments. These efforts will be crucial for optimizing its formulation, enhancing local bioavailability, and maximizing its therapeutic potential for the management of glaucoma.

In conclusion, this work demonstrates that composite liposomes with increased antioxidant capacity have been successfully synthesized, making them promising candidates as a neuroprotective therapy for glaucoma and, since oxidative stress does indeed contribute to the development and progression of the disease, antioxidants should be incorporated into treatment plans.

## Figures and Tables

**Table 1:** Antioxidant activity calculated as the inhibition percentage of DPPH radical.

Sample	Scavenger Activity (%)
Liposome	0.0
Lipoic acid solution (5 mg/mL)	5.3 ± 0.3 ***
Liposome with lipoic acid (4.03 mg/mL)	31.6 ± 0.4 ***,+++

Results are expressed as mean ± SEM. Statistical significance of the differences among was calculated by one-way ANOVA test followed by Bonferroni’s test as a post hoc test *** *p* < 0.001 vs. liposome, ^+++^ *p* < 0.001 vs. lipoic acid solution.

## Data Availability

The data presented in this study are available on request from the corresponding author.

## Abbreviations

The following abbreviations are used in this manuscript:

DPPC	1,2-dipalmitoyl-sn-glycero-3-phosphatidylcholine
DPPH•	2,2-diphenyl-1-picrylhydrazyl free radical
DLS	Dynamic Light Scattering
FTIR	Fourier transform infrared
L	Liposomes
LA	Lipoic acid
L-LA	Liposomes with lipoic acid
MTT	3-(4,5-dimethyl-thiazol-2-yl)-2,5-diphenyl-tetrazolium bromide
POPC	1-palmitoyl-2-oleoyl-sn-glycero-3-phosphatidylcholine
RGCs	Retinal ganglion cells
TEM	Transmission electron microscopy
Tm	Transition temperature
